# Variability of ChatGPT in Interpreting the Lexicon of ACR-TIRADS, EU-TIRADS, and K-TIRADS

**DOI:** 10.3390/diagnostics15212694

**Published:** 2025-10-24

**Authors:** Pierpaolo Trimboli, Amos Colombo, Lorenzo Ruinelli, Andrea Leoncini

**Affiliations:** 1 Thyroid Unit, Clinic for Endocrinology and Diabetology, Ente Ospedaliero Cantonale, 6500 Bellinzona, Switzerland; 2 Faculty of Biomedical Sciences, Università della Svizzera Italiana (USI), 6900 Lugano, Switzerland; 3 Clinical Trial Unit, Ente Ospedaliero Cantonale, 6500 Bellinzona, Switzerland; 4 Team Innovation and Research, Area ICT, Ente Ospedaliero Cantonale, 6500 Bellinzona, Switzerland; 5 Clinic for Radiology, Imaging Institute of Southern Switzerland, Ente Ospedaliero Cantonale, 6500 Bellinzona, Switzerland

**Keywords:** Thyroid, ultrasound, TIRADS, Artificial Intelligence, ChatGPT

## Abstract

**Background:** There is an ongoing project to create an international Thyroid Imaging Reporting And Data System (I-TIRADS) to harmonize the terminology of guidelines for reporting thyroid ultrasonography. As artificial intelligence (AI) has been gaining increasing attention also in the thyroid field, achieving solid information about the consistency of AI in interpreting the TIRADS terminology is relevant before the I-TIRADS is published. The present study aimed to examine the issue of AI when interpreting the TIRADS terminology to describe thyroid nodules (TNs). **Methods:** Three TIRADSs from the USA (ACR-TIRADS), Europe (EU-TIRADS), and Asia (K-TIRADS) were considered. The most popular AI, such as ChatGPT, was tested. All possible combinations of terms of the three TIRADSs were performed. **Results:** 2592 cases were included. With the ACR-TIRADS lexicon, there was a slightly significant difference between systems (*p* = 0.0494) which was attributed to variations between ACR- and EU-TIRADS (*p* = 0.0099). With the EU-TIRADS lexicon, there was a significant difference between systems (*p* < 0.0001) with a significant result between EU- and ACR-TIRADS (*p* = 0.0003). Using the K-TIRADS terminology, no significant difference was observed (*p* = 0.7954). The intraobserver agreement of ChatGPT was moderate with the best values (from 0.55 to 0.60) with the K-TIRADS lexicon. **Conclusions:** ChatGPT interprets the TIRADS lexicon but with variations when it is asked to assess TNs according to one TIRADS using the terminology of another TIRADS. Clinical operators as well as patients should also be aware of these novel data.

## 1. Introduction

Artificial intelligence (AI) has been gaining increasing attention in every field of human interest including medicine. AI is a term introduced in the 1950s to describe a branch of computer science that uses mathematical algorithms to perform tasks normally requiring human cognitive abilities [1]. In medicine, AI models have been frequently used in diagnostic imaging examinations (e.g., ultrasonography, computed tomography scan, magnetic resonance imaging, and radiography) [2]. Previous studies commonly aimed to evaluate whether AI can enhance human performance primarily in disease areas including oncology, neurology, and cardiology [3,4]. In the thyroid field, even if the issue should still be discussed, AI-based methodologies are considered promising in improving diagnostic accuracy [5]. Most medical studies tested the reliability of AI in image interpretation. Nevertheless, AI models are also conceived to read text, interpret various terminologies, and furnish potential operative suggestions after proper training [6,7].

Harmonizing the terminology of international guidelines for reporting thyroid ultrasonography (US) examination and developing a standardized lexicon, and then a joint multisociety guideline currently represents a hot topic [8]. Based on data in the literature, the systems have a high performance even though they have some differences [9]. Currently, there is an ongoing project that aims to produce a final document (the international TIRADS, I-TIRADS) endorsed by the participating societies that will replace the existing TIRADSs [8]. As this issue involves all clinical thyroidologists and AI models are accessible to medical personnel and patients, achieving solid information about the consistency of AI in interpreting the TIRADS terminology is highly relevant before the I-TIRADS is published.

This study aimed to evaluate the semantic consistency of ChatGPT when interpreting thyroid nodule descriptions across the three major TIRADS lexicons (ACR-, EU-, and K-TIRADS) [10,11,12]. The most popular AI model, such as ChatGPT, was tested. All possible combinations of terms of the three TIRADSs were performed. ChatGPT was used to classify all scenarios across the categories of the three systems, and the intraobserver agreement (IAO) was calculated.

## 2. Material and Methods

### 2.1. Conduction of Study

The present study was conducted according to STARD guidelines.

### 2.2. TIRADS Characteristics

**Figure 1:**
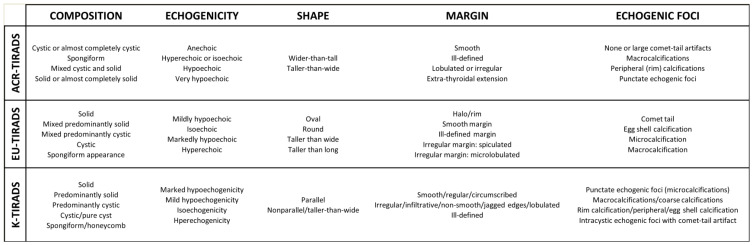
Ultrasonographic descriptors of the five ultrasound issues included in the three most diffused TIRADSs.

At present, several classification systems for the US-based risk of thyroid nodule (TN) malignancy are available with the acronym Thyroid Imaging Reporting And Data System (TIRADS). These systems aim to (1) standardize the lexicon that should be used in thyroid US reports and (2) improve the selection of TNs for biopsy to the greatest extent possible. Discrepancies are present between the three most diffused TIRADSs in the lexicon that they included (Figure 1) [10,11,12].

### 2.3. Study Design

The terminologies such as ACR-, EU-, and K-TIRADS [10,11,12] were analyzed, and their issues (composition, echogenicity, margin, shape, and echogenic foci) and the associated descriptors were extracted. All descriptors were combined with each other in all possible combinations. Initially, OpenAI’s ChatGPT (GPT-4o Mini) was asked to classify all scenarios of each TIRADS across its specific risk categories. A total of 2592 cases were included; they were synthetic combinations of descriptors and not real patients. Then, ChatGPT was asked to assess the cases reported according to the terminology of any TIRADS across the categories of the other TIRADSs. Finally, the IOA between assessments was calculated. The approach was zero-shot, meaning ChatGPT was given a task without any examples and had to respond solely based on the provided instructions. The instructions consisted of a fixed system prompt combined with a dynamic prompt detailing the target classification system and the defining nodule descriptors. The model was accessed using the OpenAI API (version 1.90.0) in Python version 3.10. The temperature of the model was set to 1, reflecting a balanced blend of creativity and coherence. Two data scientists (AC and LR) performed the analyses between May 2024 and January 2025.

### 2.4. Ethics Approval

This study did not include data from humans. Therefore, the ethics committee approval was waived.

### 2.5. Statistical Analysis

The ChatGPT assessments according to the TIRADSs were compared using the chi-square test. The IOA was estimated based on the Cohen kappa (κ), where κ indicates the strength of agreement as follows: 0–0.20, no agreement; 0.21–0.40, fair agreement; 0.41–0.60, moderate agreement; 0.61–0.80, substantial agreement; and 0.81–1.0, almost perfect agreement. Cases with different assessments were evaluated to identify descriptors associated with discordances, and all discordances of any descriptor were counted. Statistical analyses were performed using the Sklearn version 1.7.1 software package (2024, scikit-learn developers [BSD License]) or the MedCalc version 2023 software (MedCalc Software Ltd., Ostend, Belgium). Figures were created with GraphPad Prism version 7 (GraphPad Software, Boston, MA, USA).

## 3. Results

### 3.1. Study Series

According to the study design, all the descriptors of the three TIRADSs were combined to achieve all possible combinations of written vignettes. Then, the study series included 2592 cases: 512 according to ACR-TIRADS, 1600 according to EU-TIRADS, and 480 according to K-TIRADS.

### 3.2. ChatGPT Assessment of Cases According to the Three Systems

The 512 vignettes of ACR-TIRADS were classified across the categories as follows: 38 TR2, 56 TR3, 149 TR4, and 269 TR5. ChatGPT classified the 1600 cases of EU-TIRADS as 42 class 2, 141 class 3, 717 class 4, and 700 class 5. The 480 scenarios of K-TIRADS were: 37 category 2, 53 category 3, 160 category 4, and 230 category 5.

### 3.3. ChatGPT Assessment Crossing the Lexicons and the TIRADSs

**Figure 2:**
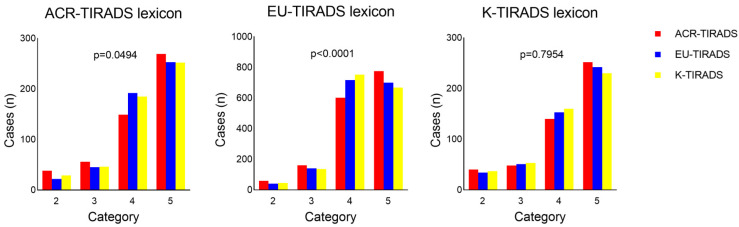
ChatGPT assessment of 2592 cases according to the three TIRADSs crossing their lexicons over each other. Legend: *p* value refers to the chi-square test among the three systems.

To explore the capability of AI in classifying TNs according to one TIRADS independent of the proposed TIRADS lexicon, ChatGPT was used to assess the cases according to any TIRADS with the terminology of the other two TIRADSs. Figure 2 shows the results. With the ACR-TIRADS lexicon, there was a slightly significant difference among the three systems (*p* = 0.0494) which was attributed to variations between ACR- and EU-TIRADS (*p* = 0.0099). However, no difference was observed between ACR- and K-TIRADS (*p* = 0.1068). With the EU-TIRADS lexicon, there was a significant difference between the three systems (*p* < 0.0001) with a remarkable result between EU- and ACR-TIRADS (*p* = 0.0003) but none between EU- and K-TIRADS (*p* = 0.5370). Using the K-TIRADS terminology, no significant difference was observed (*p* = 0.7954) (K- vs. ACR-TIRADS, *p* = 0.4399; K- vs. EU-TIRADS, *p* = 0.5811). Overall, as shown in Figure 2, the EU-TIRADS analysis yielded very strong statistical evidence while the ACR-TIRADS analysis met the 0.05 threshold and the K-TIRADS did not. Interpretation, however, should emphasize effect sizes.

The IOA of ChatGPT was calculated for any couple of assessments. As shown in Table 1, the IOA was moderate in all cases, with the best value between K- and EU-TIRADS using the K-TIRADS lexicon. The highest κ values (from 0.55 to 0.60) were observed with the K-TIRADS lexicon. The lowest κ values (from 0.41 to 0.47) were found with the EU-TIRADS lexicon. The results of the ACR-TIRADS terminology had intermediate values (from 0.48 to 0.54).

### 3.4. Analysis of the Discordant Cases

The descriptors of the three TIRADSs (see Figure 1) were analyzed to collect information about their impact on generating discordant cases. Table 2 depicts the descriptors associated with more than one discordance in the ChatGPT assessment according to the three TIRADSs.

## 4. Discussion

**Figure 3:**
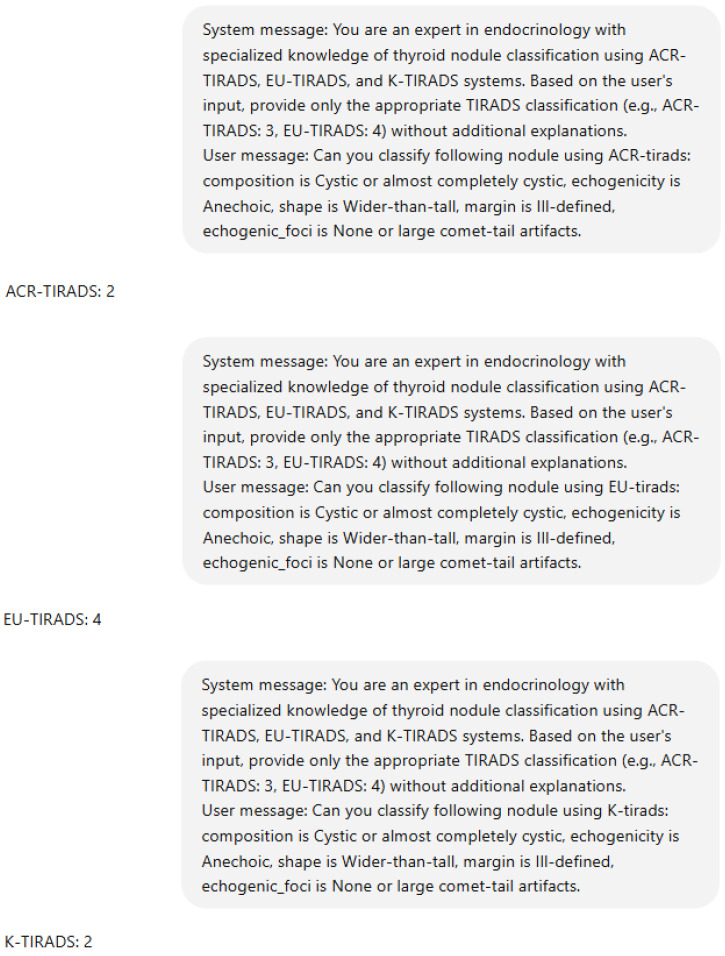
Example of a prompt submitted to ChatGPT via the OpenAI API. Legend: In this example, ChatGPT was asked to classify a scenario according to ACR-, EU-, and K-TIRADS using the terminology of ACR-TIRADS.

ChatGPT demonstrated moderate but variable consistency across TIRADS lexicons, with the best performance using K-TIRADS terminology. ChatGPT learns patterns, syntax, and semantics via self-supervised learning, thereby enabling it to perform tasks such as text generation, summary, and question answering [13]. The current study utilizes OpenAI’s ChatGPT (GPT-4o Mini) accessed via the API. The model’s context is established by a system message, specifying that it should act as an expert in endocrinology/thyroidology. This system’s message remains consistent across all scenarios. Figure 3 shows an example of the prompt used with discordant results among the three TIRADSs.

Previous studies focused on the reliability of ChatGPT in assessing the risk of TN malignancy according to US images [14]. Others have evaluated the competence and reliability of ChatGPT in thyroid cancer [15,16]. In some cases, ChatGPT results were compared with those of young clinicians or surgeons [17,18]. Finally, one very interesting study investigated the accuracy and reproducibility of ChatGPT in generating structured US reports according to TIRADS [19]. To date, no study has evaluated the ability of ChatGPT in reading texts and assessing TNs according to TIRADSs. In view of the current relevant project to create an international TIRADS using a unique lexicon, this data can be highly interesting for both clinical operators and the expert board members of I-TIRADS [8]. Thyroid clinicians, endocrine surgeons, and radiologists should be fully aware of the important role of TN reports in improving subsequent case management [20].

The current project challenged the issue of AI understanding of the TIRADS lexicon by exploring the IOA of ChatGPT when it was asked to assess TNs according to one TIRADS using the terms of other TIRADSs. The results are highly interesting for medical professionals and AI model developers. Considering that AI is easily accessible to everyone, patients with thyroid conditions are further stakeholders [6,7,21]. The findings of the current study can be summarized as follows: First, when ChatGPT was asked to classify all the possible TN scenarios of the three major TIRADSs, it tended to assess cases across their higher-risk categories. As the study did not include a human reference to assess the capability of ChatGPT in assessing the risk of TN malignancy, this finding cannot be validated. However, these data suggest the need for specific studies that can verify whether the interpretation of ChatGPT is generally in favor of high-risk assessment. Second, when ChatGPT was required to classify TNs according to one TIRADS using the lexicon of the other two TIRADSs, a significantly different assessment result was observed only between ACR- and EU-TIRADS with both the ACR- and EU-TIRADS terminology. These data have a high relevance, showing that the K-TIRADS lexicon is easily interpreted by ChatGPT when it is asked to assess TNs across the categories of the other systems. However, we should be taken into account that this finding may reflect lexical simplicity or overlap rather than a true semantic advantage. Third, the analysis of the ChatGPT IOA had the highest values with the K-TIRADS lexicon, intermediate κ values with the ACR-TIRADS terminology, and the lowest performance with the EU-TIRADS. This represents the most powerful study result and demonstrates that the terminology included in the K-TIRADS is easily interchangeable among the three systems. Fourth, the number of descriptors associated with the higher frequency of discordances between TIRADS was lower in K-TIRADS than in the other two systems. These data should reflect that the Korean terminology is, at least partially, overlapping with that of the other two systems. The higher ChatGPT interpretability of K-TIRADS compared with ACR-TIRADS and EU-TIRADS suggests that its criteria may be more intuitive and easier to apply in clinical practice. This could enhance diagnostic consistency among less experienced operators. From an educational perspective, the terminology of K-TIRADS may facilitate training and improve adherence to guidelines.

Based on the results of this study, a clinically oriented discussion can be addressed. During the conceptualization of the study design, the authors of this study, which are endocrinologists, radiologists, and computer scientists initially hypothesized that the most popular terminology such as ACR-TIRADS would have the best results. ACR-TIRADS is the only point-based system and is the easiest system for non-medical personnel who are required to assess TNs [22]. However, to completely explore the matter and prevent potential biases due to the selected cases, ChatGPT was queried about all possible combinations of the descriptors of the three systems. Surprisingly, K-TIRADS had the best performance. Indeed, ChatGPT assessed TNs across the K-TIRADS categories using the lexicon of the other TIRADSs without significant differences with respect to the original TIRADS of that lexicon. In addition, K-TIRADS allowed ChatGPT to assess TNs according to any TIRADS with the highest IOA lexicon. The reasons for these data can vary. The last version of K-TIRADS was published in 2021, which is four years later than that of ACR- and EU-TIRADS. Then, it may be conceived on the basis of the previous systems and the literature. The lexicon of K-TIRADS could have been developed according to the other systems, thereby representing their advancement. In addition, as shown in Figure 1, the descriptors of K-TIRADS can be reported in multiple synonyms (e.g., the “abnormal” nodule’s margin could be described as irregular or infiltrative or jagged edges or lobulated or ill-defined) that can facilitate the interpretation of AI models. Other explanations may be possible. According to this finding, it should be affirmed that the terminology included in the Korean system may be the most generalizable, at least for AI. The next steps of the project that can develop an I-TIRADS should consider these novel results.

The current study had several limitations and strengths that should be addressed. First, the study relies on a single model that is not the state-of-the-art model. The study aimed at demonstrating the potential of technology rather than to benchmark it against the top-performing Large Language Models. For this reason, we selected GPT-4o-mini, which generally exhibits fewer hallucinations and strong performance while being more cost-efficient. Moreover, it benefits from training with Reinforcement Learning from Human Feedback (RLHF) enhancing its reliability (OpenAI, GPT-4o mini: advancing cost-efficient intelligence, https://openai.com/index/gpt-4o-mini-advancing-cost-efficient-intelligence/?utm_source=chatgpt.com (accessed on 18 July 2024)). Second, an explanation for the model has not been addressed, even though a linguistic model could provide classifications along with explanations that are understandable to humans, thereby offering transparency in the decision-making process. Third, a reference standard for evaluating the reliability of ChatGPT in understanding TIRADS lexicons was not included. However, whether ChatGPT is accurate in assessing the risk of TNs is not a matter of this study. Fourth, the risk of malignancy estimated in any category of any TIRADS is not always overlapping with the same numerical category of the other TIRADSs [23]. However, there is evidence showing that the accuracy of TIRADSs in assessing TNs across their numerical categories is almost similar [24]. Fifth, all findings of the ChatGPT assessment may vary according to the modality we use to ask it to assess TNs. However, this aspect cannot be evaluated otherwise. Fourth, K-TIRADS was initially published in 2017 and was later revised in 2021. Then, its results can be biased by the learning of ChatGPT from both documents and the related literature. The authors of this research did not investigate this issue to avoid confounding data whose association with the first or the second version of K-TIRADS could not be distinguished.

## 5. Conclusions

In conclusion, ChatGPT interprets the lexicon of TIRADS and assesses TNs across their risk categories. However, this capability is not always maintained when ChatGPT is asked to assess TNs according to one TIRADS using the terminology of another TIRADS. The important project involving the development of I-TIRADS should consider the findings of the current study. Clinicians and researchers should be aware of these findings when considering AI tools for nodule assessment, as they have potential implications for patient communication and education.

## Figures and Tables

**Table 1:** Results of the ChatGPT intraobserver agreement in the assessment of TNs according to different lexicons. Legend: The values in the table were expressed as Cohen kappa (κ).

	ACR-TIRADS Lexicon	EU-TIRADS Lexicon	K-TIRADS Lexicon
ACR vs. EU	0.50	0.47	0.55
ACR vs. K	0.54	0.41	0.55
K vs. EU	0.48	0.43	0.60

**Table 2:** Analysis of the descriptors with the highest frequency of discordance between the TIRADS. Legend: The table shows the results of the descriptors with the number of discordances between couples of TIRADSs that are higher than the number of discordant cases.

Lexicon	Descriptor	Cases	Discordances			
			Number	ACR vs. EU	ACR vs. K	EU vs. K
ACR-TIRADS	Cystic or almost completely cystic	128	163	52	54	57
	Smooth	128	147	50	51	46
	Spongiform	128	143	47	46	50
	Anechoic	128	138	48	47	43
EU-TIRADS	Spongiform appearance	320	459	158	140	161
	Smooth margin	320	372	124	130	118
	Ill-defined margin	320	371	101	133	137
	Cystic	320	364	113	131	120
	Halo/rim	320	357	121	123	113
	Round	400	442	139	152	151
	Egg shell calcification	400	429	139	148	142
	Hyperechoic	400	427	139	157	131
	Isoechoic	400	423	130	148	145
K-TIRADS	Smooth/regular/circumscribed	160	178	69	58	51
	Spongiform/honeycomb	96	105	33	41	31

## Data Availability

The dataset of the study is available from the corresponding author upon reasonable request.
